# The Therapeutic Effects of a Traditional Chinese Medicine Formula Wuzi Yanzong Pill for the Treatment of Oligoasthenozoospermia: A Meta-Analysis of Randomized Controlled Trials

**DOI:** 10.1155/2018/2968025

**Published:** 2018-01-17

**Authors:** Ming Peng Zhao, Xiao Shi, Grace Wing Shan Kong, Chi Chiu Wang, Justin Che Yuen Wu, Zhi Xiu Lin, Tin Chiu Li, David Yiu Leung Chan

**Affiliations:** ^1^Department of Obstetrics and Gynecology, Prince of Wales Hospital, The Chinese University of Hong Kong, Sha Tin, Hong Kong; ^2^Department of Medicine and Therapeutics, Prince of Wales Hospital, The Chinese University of Hong Kong, Sha Tin, Hong Kong; ^3^School of Chinese Medicine, The Chinese University of Hong Kong, Sha Tin, Hong Kong; ^4^Hong Kong Institute of Integrative Medicine, Faculty of Medicine, The Chinese University of Hong Kong, Sha Tin, Hong Kong

## Abstract

Oligoasthenozoospermia is a crucial factor in male infertility. Wuzi Yanzong (WZYZ) pill is a popular traditional Chinese medicine (TCM) formula which has been used for male infertility treatment for years. However, its effects on semen quality remain controversial. We conducted a preregistered meta-analysis to assess the effect of WZYZ pill for the therapeutic effects on oligoasthenozoospermia. Five randomized controlled trials including 960 participants were selected from databases of domains in North-East Asian regions, PubMed, Embase, and Cochrane Library. WZYZ pill group yielded a greater mean increment on sperm concentration (5 trials: MD 5.99, 95% CI 2.12–9.85, *P* = 0.002), sperm motility (5 trials: MD 4.57, 95% CI 0.47–8.68, *P* = 0.03), sperm morphology (2 trials: MD −1.93, 95% CI −4.87–1.01, *P* = 0.20), activity of acrosomal enzyme (2 trials: MD 28.27, 95% CI 12.41–44.14, *P* < 0.01), volume of semen (2 trials: MD 0.56, 95% CI 0.21–0.91, *P* = 0.002), and a decrement of sperm DNA fragmentation index (2 trials: MD −3.82, 95% CI −6.45–−1.19, *P* = 0.004). However, qualities of selected studies were generally unsatisfactory, and there was inherent heterogeneity among some of the outcomes. Despite these limitations, the WZYZ pill improved sperm quality by improving several semen parameters and decreasing DNA damage in oligoasthenozoospermia patients.

## 1. Introduction

It is reported that 15% of couples suffer from fertility problems and nearly half of them are caused by male factors [1, 2]. As one of the male infertility factors, Oligoasthenozoospermia is defined as the total number or concentration of spermatozoa and percentage of progressively motile spermatozoa, below the lower reference limits (total number < 33 × 10^6^ per ejaculate; concentration < 12 × 10^6^ per ml; progressively motile < 31%) [3]. More than 40% of infertile men are diagnosed with oligoasthenozoospermia, and their poor sperm quality is considered as one of the major causes of infertility [4]. Improving the sperm quality in these patients is one of the main targets of male infertility therapy. However, effective pharmaceutical treatments for oligoasthenozoospermia are lacking [5]. In recent years, attentions have been drawn to the use of alternative complementary medicine including traditional Chinese medicine.

From ancient times to the present, Chinese people have been using the combination of herbs to improve human fertility. Nowadays, some researchers have reported positive effects of different Chinese herbal formulas on male fertility, like* Cordyceps militaris* [6], Shao-Fu-Zhu-Yu-Tang [7], and Ginseng [8] in a range of biochemical and clinical studies.

The Wuzi Yanzong (WZYZ) pill is one of the most commonly prescribed Chinese herbal formulas for the treatment of male infertility. It was first recorded in the book called “Xuan Jie Lu” in AD 733 and spread around North-East Asia over the next thousand years [9]. This herbal formula consists of five herbal seeds:* Cuscutae Chinensis Semen, Lycii Fructus, Rubi Fructus, Schizandrae Fructus, and Plantaginis Semen*. According to the theory of traditional Chinese pharmacopoeia, this formula has benefits on both male fertility and kidneys' function. This formula was designed to treat infertility due to kidney deficiency manifesting as impotence, premature ejaculation, and poor quality of semen and has been appraised since ancient time as “The Number One Herbal Formula for Assisting Fertility” [9].

Though the WZYZ pill has been used as a treatment of male infertility for a long time in North-East Asia, the efficacy of WZYZ pill in treating male infertility remains a matter of debate and meta-analysis in this field is scarce. Thus, this meta-analysis aims to evaluate the therapeutic effect of the WZYZ pill in patients with oligoasthenozoospermia from a rigorous selection of randomized controlled trials (RCT).

## 2. Materials and Methods

### 2.1. Search Strategy

A prospective protocol of objectives, literature search strategies, inclusion and exclusion criteria, outcome measurements, and methods of statistical analysis were prepared a priori according to the Preferred Reporting Items for Systematic Reviews and Meta-analysis (PRISMA) of Observation Studies in Epidemiology recommendations for study reporting [10, 11]. We registered this meta-analysis at International Prospective Register of Systematic Reviews (number CRD42017059605) [10].

Vitamins C and E are commonly used as control interventions for sperm quality studies [12, 13]; RCTs [14–16] have confirmed these vitamin controls are the equivalent of placebos. Thus, we included all the published, relevant randomized controlled trials of the WZYZ pill versus placebo or vitamin control up to March 17, 2017, without language restriction to conduct the meta-analysis of the sole effect of the WZYZ pill. As the WZYZ pill has been used for more than thousand years by Chinese people, it has also been used by other Asian countries. Therefore, our searched databases included electronic databases of the most domain from North-East Asian regions. The databases we searched in this review included China National Knowledge Infrastructure (China Mainland), Wangfang Database (China Mainland), Index to Chinese Periodicals of Hong Kong (Hong Kong), Macau Periodical Index (Macau), Airitilibrary (Taiwan), Korean Journal Publishing Service (Korea), Korean Studies Information Service System (Korea), Medical Online (Japan), PubMed (Global), Embase (Global), and Cochrane Central Register of Controlled Trials Online (Global). The detail of search strategies is posted in PROSPERO (number CRD42017059605). In brief, different search strategies were combined as follows: for the Chinese Mainland databases, subject terms as “Wuzi Yanzong,” “oligospermia,” “asthenozoospermia,” and “randomized” were used. For the other North-East Asian databases, subject term “Wuzi Yanzong” in Mandarin, Cantonese, Taiwanese, and Japanese version was used. “Ojayeonjonghwan” and “KH-204,” the Korean versions of the Wuzi Yanzong pill, were also used to search in both the Korean and Global database. For the Global database, the subject terms “Wuzi Yanzong,” “oligospermia,” “asthenozoospermia,” and “randomized controlled trials” were searched in title/abstract. Manual searches of the reference lists of all retrieved studies, review articles, and conference abstracts were supplemented with cyber-search. When multiple reports describing the same study population were published, the most recent or complete report was used.

### 2.2. Eligibility Criteria

We regarded studies as eligible for inclusion if (1) they were RCTs, (2) performed on adults with oligoasthenozoospermia, (3) treated with WZYZ pill (regardless of classical or modified), (4) compared with placebo or vitamin control, (5) had at least three months' duration of intervention, and (6) reported sperm analysis outcomes. Exclusion criteria were as follows: (1) observational and retrospective studies, (2) no control group, (3) studies with a comparison between WZYZ pill and other TCM therapies without placebo or vitamin control, and (4) animal experimental studies.

### 2.3. Study Selection and Data Extraction

Two independent investigators (Zhao and Shi) reviewed the study titles and abstracts, and studies that satisfied the inclusion criteria underwent full-text assessment according to the PRISMA recommendations [10]. Any disagreement was resolved by the third adjudicating author (Chan). We extracted the following data from each selected study: authors, year of published, study location, total number of participants, participants' mean age, intervention, trial duration, method of measurement, type of control group, change of sperm concentration, sperm motility, normal rate of sperm morphology, semen volume, sperm DNA fragmentation index, and activity of acrosomal enzyme after treatment. The primary outcomes were sperm concentration, sperm motility, and the normal rate of sperm morphology. The secondary outcomes were the volume of semen, sperm DNA fragmentation index, and the activity of acrosomal enzyme.

### 2.4. Quality Assessment

Two independent investigators (Zhao and Shi) assessed the methodological quality of RCTs according to the Cochrane risk of bias tool [17]. The methodological issues related to the quality of RCT were the generation of treatment allocation, concealment of treatment, blinding, incomplete outcome analysis, nonselective outcome reporting, and other potential risks of bias. Any disagreement was resolved by the third adjudicating author (Chan).

### 2.5. Statistical Analysis

This meta-analysis was performed using Review Manager 5.2 (Cochrane Collaboration, Oxford, UK). Since all the data extracted from studies were continuous variables across the same scales, we used the mean difference (MD) for comparison. All results were reported with 95% confidence intervals (CIs).

We calculated pooled estimates of mean differences in sperm concentration, sperm motility, normal rate of sperm morphology, volume of semen, sperm DNA fragmentation index, and activity of acrosomal enzyme using the technique described by Hozo et al. [18]. Statistical heterogeneity between studies was assessed using the Cochrane *Q* test [19] with significance set at *P* < 0.10. We also performed *I*^2^ testing [20] to evaluate the magnitude of heterogeneity between studies, with values greater than 50% regarded as being indicative of a moderate-to-high heterogeneity statistic. Random-effects model (DerSimonian-Laird method) was used if there was heterogeneity between studies; otherwise, the fixed-effects model was used [17].

Subgroup analyses were performed to compare different semen analysis methods. In the meta-analysis of outcomes that were common to all studies, we performed preplanned sensitivity analyses restricted to trials that compared a manual method to other methods. Since the selected studies are limited, we did not screen publication bias.

## 3. Results

**Figure 1:**
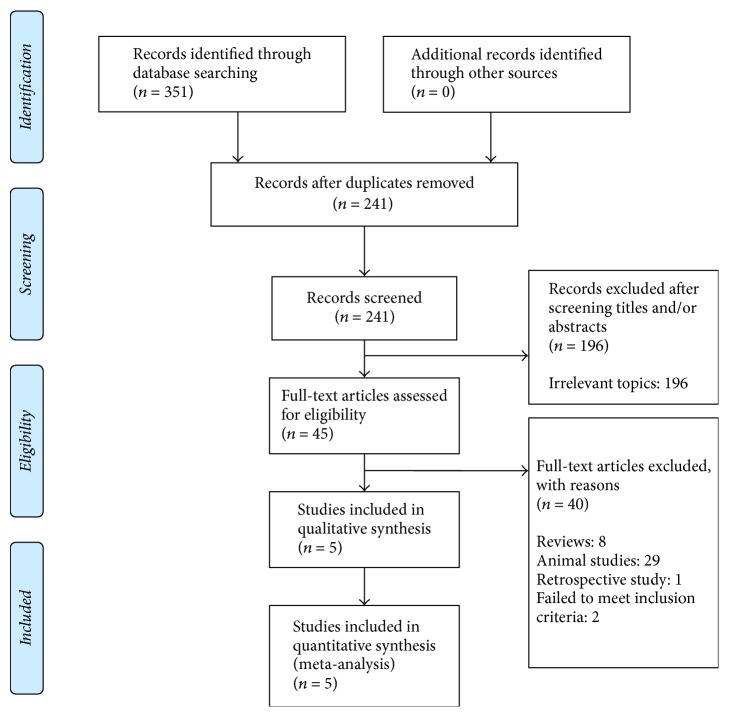
Flow chart of study selection.

The initial search identified 351 citations (no additional records were identified through other resources), of which 110 duplicated publications were removed. One hundred and ninety-six citations of irrelevant topics were excluded following a screening of the titles and abstracts, and 45 manuscripts were retrieved and reviewed in full. Of the 45 manuscripts examined in full, 40 were excluded (8 reviews, 29 animal studies, one retrospective study, and two studies failed to meet the inclusion criteria). Five RCTs including 960 participants (509 participants for WZYZ pill treatment and 451 participants for placebo or vitamin control treatment) fulfilled the predefined inclusion criteria and were included in the final analysis (Figure 1). Examination of the references listed for these studies did not yield any further studies for evaluation. Agreement between the two reviewers was 92% for study selection and 80% for quality assessment of trials. The five trials [21–25] were all published between 2010 and 2017 (Table 1). Three trials [21, 22, 25] compared WZYZ pill with vitamin control, and the other two trials [23, 24] compared WZYZ pill with placebo control. Three trials [21, 22, 25] measured sperm parameters by manual method while two trials [23, 24] used computer-assisted semen analysis (CASA) methods. All included studies claimed their patients in each group had the same baseline of age, infertility duration, and sperm parameters. It should be mentioned that most of the chosen studies lacked the sperm parameters before WZYZ pill treatment, so this meta-analysis delineated the outcomes after treatment. The characteristics and reported sperm parameters of selected studies are shown in Table 1.

**Figure 2:**
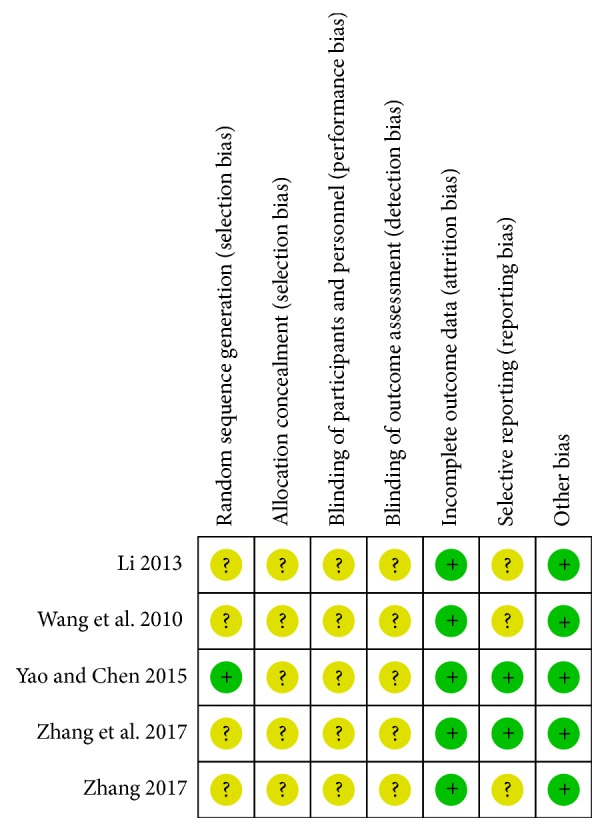
Summary of study quality assessment. Green circle indicated low risk of bias while yellow circle indicated unclear risk of bias.

The quality of all of the included studies was generally unsatisfactory or remained unclear because most of them had a high portion of the unclear risk of biases. All of the included studies were limited to make a judgment on “high” or “low” risk, and their quality assessments are largely in “unclear risk of bias”. Although five studies claimed themselves as randomized trials, only one study [23] demonstrated the detail of randomization. None of the studies gave the details of allocation concealment, blinding of participants and personnel, and the blinding of outcome assessment. Two studies [23, 24] took the WZYZ pill as one of the control arms compared with other drug treatment effects, which we considered were of low risk of selective reporting bias. The rate of losing follow-up patients was low in all studies, which meant the risk of attrition bias was low. We believe there were no other biases in each study. The summary of the risk of bias is shown in Figure 2.

### 3.1. Primary Outcomes

The results of meta-analysis comparison of WZYZ pill and placebo or vitamin control are shown in Table 2.

**Figure 3:**
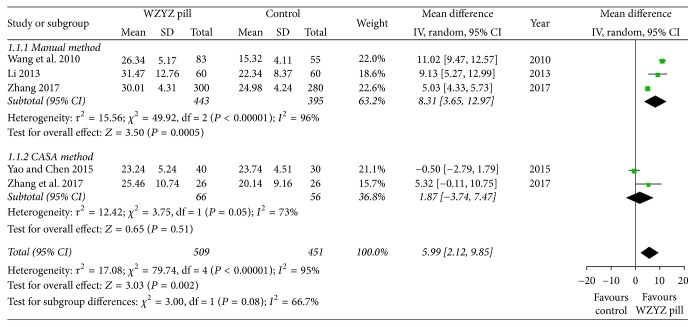
Forest plot and meta-analysis of sperm concentration.

In a pooled analysis of all five trials, the combination of sperm concentration led to a mean greater increment in the WZYZ pill group compared to the placebo or vitamin control (MD 5.99, 95% CI 2.12–9.85, *P* = 0.002), with high statistical significance heterogeneity (*Q* < 0.01, *I*^2^ = 95%) (Figure 3). Further subgroup analysis in comparing WZYZ pill with manual method (3 trials 838 participants) showed higher statistically significant results (MD 8.31, 95% CI 3.65–12.97, *P* < 0.01) but also higher heterogeneity (*I*^2^ = 96%) (Figure 3). When comparing the CASA method (2 trials 122 participants), the result of WZYZ pill treatment became compromised (MD 1.87, 95% CI −3.74 to 7.47, *P* = 0.51), with high heterogeneity (*Q* = 0.05, *I*^2^ = 73%) (Figure 3).

**Figure 4:**
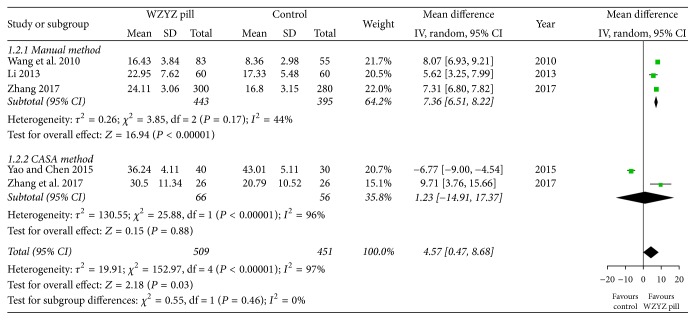
Forest plot and meta-analysis of sperm motility.

All studies reported the outcome of sperm motility. Pooled analysis of the five studies showed a higher increment of sperm motility in the WZYZ pill group compared to that of the placebo or vitamin control (MD 4.57, 95% CI 0.47–8.68, *P* = 0.03), with high statistical heterogeneity (*Q* < 0.01, *I*^2^ = 97%) (Figure 4). Further subgroup analysis in comparing WYZY pill with manual method (3 trials 838 participants) showed a higher statistically significant result (MD 7.36, 95% CI 6.51–8.22, *P* < 0.01) with low heterogeneity level (*Q* = 0.17, *I*^2^ = 44%) (Figure 4). When comparing with the CASA method (2 trials 122 participants), the result of the WZYZ pill treatment became compromised (MD 1.23, 95% CI −14.91–17.37, *P* = 0.88), with high heterogeneity (*Q* < 0.01, *I*^2^ = 96%) (Figure 4).

**Figure 5:**
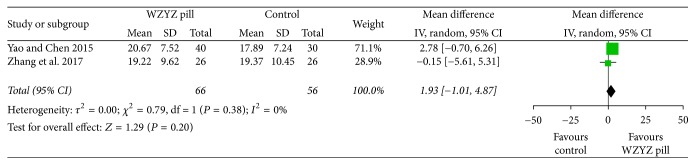
Forest plot and meta-analysis of sperm morphology.

The two studies [23, 24] reported the outcome of sperm morphology. In a pooled analysis of these two trials, no significant difference was found between the two groups regarding the normal rate of sperm morphology (MD 1.93, 95% CI −1.01–4.87, *P* = 0.20), without heterogeneity (*Q* < 0.38, *I*^2^ = 0%) (Figure 5).

### 3.2. Secondary Outcomes

**Figure 6:**
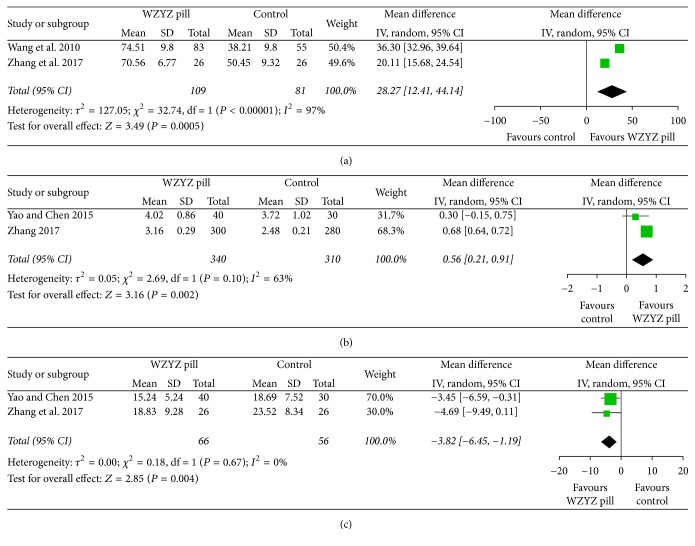
Forest plot and meta-analysis of secondary outcomes. Outcomes assessed are (a) activity of acrosomal enzyme, (b) semen volume, and (c) DNA fragmentation index.

Two studies [21, 24] reported the results of the activity of the acrosomal enzyme. In a pooled analysis of these two trials, the combination of the acrosomal enzyme activity led to a greater mean increment in the WZYZ pill group than in the control group (MD 28.27, 95% CI 12.41–44.14, *P* < 0.01), with high heterogeneity (*Q* < 0.01, *I*^2^ = 97%) (Figure 6(a)). Two studies [23, 25] reported the volume of semen after treatment. The pooled analysis of these two trial showed a statistical significance between the WZYZ pill group and control group (MD 0.56, 95% CI 0.21–0.91, *P* = 0.002), with moderate-to-high heterogeneity (*Q* = 0.1, *I*^2^ = 63%) (Figure 6(b)). Only two studies compared with placebo [23, 24] reported the outcome of sperm DNA fragmentation index. In a pooled analysis of these two trials, our results showed that, in the WZYZ pill group, sperm DNA fragmentation index led to a mean greater reduction than in the control group (MD −3.82, 95% CI −6.45–1.19, *P* = 0.004), without heterogeneity (*Q* = 0.67, *I*^2^ = 0%) (Figure 6(c)).

## 4. Discussion

This meta-analysis used rigorous eligibility criteria for study selection and resulted in the analysis of five RCTs (including 960 patients) to compare the efficacy of the WZYZ pill with placebo or vitamin control. Our results showed that the WZYZ pill did significantly increase the sperm concentration and improve the sperm motility. Furthermore, compared with placebo, the WZYZ pill offered a greater reduction of sperm DNA fragmentation index and improved both the activity of acrosomal enzyme and the volume of semen. These data thus support the possible use of the WZYZ pill as a therapeutic strategy that can improve sperm quality in patients with oligoasthenozoospermia.

### 4.1. Sperm Concentration

The editorial committee of WHO semen examination manual [3] considered that although concentration could not thoroughly reflect the testicular function, concentration is closely related to fertilization rate and pregnancy rates, and it is, therefore, one of the most important diagnostic criteria of oligoasthenozoospermia. Our pooled data of sperm concentration results indicated that the WZYZ pill increased the sperm concentration compared to placebo or vitamin control. However, this result should be viewed with caution as the heterogeneity of this comparison was high. In order to analyze the cause of heterogeneity, we conducted subgroup analyses. However, none of the subgroups significantly reduced their heterogeneity, which indicated that the heterogeneity was not caused by clinical heterogeneity origin patinating from different examination methods (manual or CASA) but other factors. Moreover, participants in these RCT studies were mainly from South China [21–23, 25], so the study population was relatively consistent. We supposed the heterogeneity might originate from other factors such as quality control. Quality control is a vital issue in the proficiency of sperm concentration and motility measurement across different andrology labs. The WHO guideline [3] emphasized that the sperm concentration is easily affected by dilution, sperm collection, and the patients' clinical status. Nevertheless, none of the manual method studies reported their quality control procedures for semen analysis, which could be a potential risk of detection bias. Therefore, heterogeneity might result from the diversity of manual measurement methods.

The effect of the WZYZ pill on sperm concentration was supported by recent studies on a molecular scale [26]. Xu and her colleagues found that the WZYZ pill had the ability to improve spermatogenesis by modulating the secretory function of sertoli cells, which might be one of the mechanisms underlying the increment of sperm concentration by WZYZ pill therapy.

### 4.2. Sperm Motility

The extent of progressive sperm motility is directly related to pregnancy rates [3], especially in subfertile couples. As it is shown in Figure 4, our study found that the WZYZ pill appears to improve sperm motility. We further conducted subgroup analysis and found that the heterogeneity decreased in the manual semen analysis subgroup compared to the total pooled data. We speculated that the high heterogeneity of total pooled data derived from the difference between CASA and manual analysis methods. Lammers and his colleagues [27] had compared the results between manual and computer-assisted methods on sperm motility and found the manual motility results were significantly higher than the computer-assisted results primarily in the severe oligozoospermic group. Although the result from the manual method subgroup gave a robust finding to support the effect of WZYZ pill on the sperm motility improvement, we cannot rule out that the significance between groups was due to the individual differences between andrologists because a qualified external QA system had not been mentioned in those studies. It should be noted that two selected studies [22, 25] only reported the rate of Grade A sperm motility while the other three reported the rate of Grade A + B of sperm motility. The difference of parameters reported in these studies was due to the difference editions of WHO manual. In the 4th edition of WHO laboratory manual [28], sperm motility is divided into four grades (A, B, C, and D) while in the latest version [3], sperm motility is described by progressive motility (by combining Grade A + B), nonprogressive motility (equivalent to Grade C), and immotility (equivalent to Grade D). For this reason, we considered it would not introduce clinical heterogeneity when pooling the results of Grade A reported studies and Grade A + B reported studies. The other semen analysis methods discussed in this meta-analysis are consistent in both editions.

### 4.3. Morphology and DNA Fragmentation Index

Evidence supporting the relationship between the percentage of normal forms and fertilization rates makes sperm morphology an important parameter to assess a patient's sperm quality [3]. In this meta-analysis, only two studies presented their data on the normal rate of sperm morphology. The pooled data showed no statistical difference between the WZYZ pill and placebo or vitamin control. However, to our surprise, the DNA fragmentation index outcome from the same studies suggested that the WZYZ pill reduced DNA fragmentation index without heterogeneity. A recent study supported this finding on a molecular scale; Ji and his colleagues [29] found that WZYZ pill treatment enhanced the expression of PCNA protein that ubiquitinated and was involved in the RAD6-dependent DNA repair pathway, resulting in reduced DNA damage in rats.

### 4.4. Other Outcomes

During the process of insemination, acromosal enzymes break down oocyte's zona pellucida, allowing sperm penetrate into the oocyte. The activity of the acrosomal enzyme is an indicator to detect the fertilization ability of sperm [3]. In this review, only two studies presented the outcomes of the activity of the acrosomal enzyme. The pooled data showed a statistical difference between the WZYZ pill and placebo or vitamin control without heterogeneity.

Regarding the outcome of semen volume, only two studies presented this parameter; thus the statistical effect was compromised. Firstly, the pooled data's heterogeneity was at a moderate-to-high level. Secondly, the number of studies was limited, and we could not perform any heterogeneity analysis on it. Thirdly, the weight of these two studies was imbalanced: while Yao and Chen's study [23] had just 70 participants in total, Zhang's study [25] included 300 participants per group and had a lower deviation of semen analyses results. Therefore, the incorporation of pooled data mainly showed the result of Zhang's study [25].

This meta-analysis showed the WZYZ pill had positive effects on the treatment of oligoasthenozoospermia. Conversely, a recent meta-analysis [30] (including 652 participants) that compared the effect of the WZYZ pill with other different TCM demonstrated a different result. They claimed that their incorporated data showed the WZYZ pill had no definite curative effect on sperm concentration, sperm motility, as well as the treatment of male infertility. We analyzed the controversial results between An and Zou's meta-analysis [30] and the present study. We found that their meta-analysis compared the WZYZ pill with other different TCMs; their study did not include any control group without any TCM treatment so the analysis could not distinguish whether WZYZ incurs any effect or not. In other words, their study cannot evaluate the sole effect of the WZYZ pill and had no sufficient data/evidence to give a conclusive result that the WZYZ pill did not show a curative effect on the treatment of oligoasthenozoospermia. Furthermore, when specifying drug interventions, factors such as the drug preparation, route of administration, dose, duration, and frequency should be considered [17]. In An and Zou's meta-analysis, most of its selected studies took WZYZ pill as a control group rather than a study group, but An and Zou converted them for their meta-analysis. Therefore their “control group” consisted of different TCMs and might have inevitably introduced inestimable clinical heterogeneity in the variation of interventions even though the statistical heterogeneity between studies was low.

The present meta-analysis, to be fair, also has the following limitations that should be highlighted here. (1) Although we set up relatively rigorous criteria of study selection, the final analysis included studies which still have a high risk of bias due to their lack of details on the process of randomization. (2) The number of selected studies was limited, so we cannot estimate the publication bias, which may compromise the accuracy of this study. (3) Many outcomes except for sperm concentration and sperm motility were limited to two or three studies, and their result of data incorporation was small. (4) Different authors used different methods to perform sperm analysis, and though we used subgroup analysis to decrease statistical heterogeneity, the study heterogeneity of various methods may still exist and compromise the results. (5) All the selected studies claimed that the baseline of their participants had no significant difference and most of the chosen studies lacked the recording of sperm parameters before WZYZ pill treatment. Therefore, this meta-analysis delineated the outcomes purely after treatment only. The lack of a comparison between sperm parameters before and after treatment might compromise the conclusion of the sole effect of the WZYZ pill. Finally, since the participants of selected studies were mainly from South China, the result of this meta-analysis may not apply to other ethnic groups.

## 5. Conclusions

This study is the first registered TCM meta-analysis that has investigated the electronic resources of the most popular domains in the North-East Asian region and has evaluated the effect of the WZYZ pill in oligoasthenozoospermia using rigorous eligibility criteria. Our results indicate that the WZYZ pill could well be a curative treatment in patients with oligoasthenozoospermia by increasing sperm concentration, improving sperm motility, enhancing the activity of the acrosomal enzyme, and decreasing sperm DNA fragmentation index. However, the inherent limitations of the included studies prevent us from reaching definitive conclusions. Well planned double-blinded RCTs that fulfill the CONSORT of RCT are needed to fully interrogate the effect of the WZYZ pill and provide more convincing evidence.

## Figures and Tables

**Table 1:** Characteristics of included studies.

Author	Year	Region	Treatment duration	Interventions	Participants number	Mean age	Baseline comparable	Methods	Reported sperm parameters					
									Con	Mot	Mph	Acr	Vol	DFI
Yao and Chen	2015	SC	3 months	K versus W versus P	110 (40/40/30)	31.25/32.11/31.74	√	CASA	√	√	√	√	X	√
Zhang et al.	2017	NEC	3 months	G versus W versus P	84 (32/26/26)	29.4/29.6/28.3	√	CASA	√	√	√	√	√	X
Zhang	2017	SC	3 months	W versus V	580 (300/280)	30.9/31.1	√	Manual	√	√^*∗*^	X	X	X	√
Li	2013	SC	3 months	W versus V	120 (60/60)	29.9/30.8	√	Manual	√	√^*∗*^	X	X	X	X
Wang et al.	2010	SC	3 months	W versus V	138 (83/55)	30.3/32.4	√	Manual	√	√	X	X	√	X

Con = Sperm Concentration; Mot = Sperm Motility; Mph = Sperm Mophorlogy; Acr = Activity of Acrosomal Enzyme; DFI = DNA Fragmentation Index; SC = South China; NEC = North-east China; W = Wuzi Yanzong pill; P = Placebo; V = Vitamin control; K = Kidney Essence Granule; G = Gonadotropins; CASA = computer-assisted semen analysis; Asterisk means the study only reported Grade A parameters of sperm motility.

**Table 2:** Results of meta-analysis comparison of WZYZ pill and placebo or vitamin control.

Outcome measures	Studies numbers	WZYZ pill participant numbers	Control participants numbers	MD (95% CI)	*P* value	Study heterogeneity			
						*X* ^2^	df	*I* ^2^, %	*P* value
Sperm concentration	5	509	451	5.99 [2.12, 9,85]	0.002	79.74	4	95	<0.00001
Sperm mobility	5	509	451	4.57 [0.47, 8.68]	0.03	152.97	4	97	<0.00001
Sperm morphology	2	66	56	−1.93 [−4.87, 1.01]	0.20	0.79	1	0	0.38
Sperm DFI	2	66	56	−3.82 [−6.45, −1.19]	0.004	0.18	1	0	0.67
Activity of acrosomal enzyme	2	109	81	28.27 [12.41, 44.14]	0.0005	32.74	1	97	<0.00001
Sperm volume	2	340	310	0.68 [0.64, 0.72]	<0.00001	2.69	1	63	<0.1

WZYZ = Wuzi Yanzong; MD = mean difference; df = degree of freedom; CI = confidence interval; DFI = DNA fragmentation index.
