# Corrigendum to “The Therapeutic Effects of a Traditional Chinese Medicine Formula Wuzi Yanzong Pill for the Treatment of Oligoasthenozoospermia: A Meta-Analysis of Randomized Controlled Trials”

**DOI:** 10.1155/2018/6109104

**Published:** 2018-06-21

**Authors:** Ming Peng Zhao, Xiao Shi, Grace Wing Shan Kong, Chi Chiu Wang, Justin Che Yuen Wu, Zhi Xiu Lin, Tin Chiu Li, David Yiu Leung Chan

**Affiliations:** ^1^Department of Obstetrics and Gynecology, Prince of Wales Hospital, The Chinese University of Hong Kong, Sha Tin, Hong Kong; ^2^Department of Medicine and Therapeutics, Prince of Wales Hospital, The Chinese University of Hong Kong, Sha Tin, Hong Kong; ^3^School of Chinese Medicine, The Chinese University of Hong Kong, Sha Tin, Hong Kong; ^4^Hong Kong Institute of Integrative Medicine, Faculty of Medicine, The Chinese University of Hong Kong, Sha Tin, Hong Kong

In the article titled “The Therapeutic Effects of a Traditional Chinese Medicine Formula Wuzi Yanzong Pill for the Treatment of Oligoasthenozoospermia: A Meta-Analysis of Randomized Controlled Trials” [1], there was an error in the Introduction, where the sentence “As one of the male infertility factors, Oligoasthenozoospermia is defined as the total number or concentration of spermatozoa and percentage of progressively motile spermatozoa, below the lower reference limits (total number < 33 × 10^6^ per ejaculate; concentration < 12 × 10^6^ per ml; progressively motile < 31%) [3]” should be corrected to “As one of the male infertility factors, oligoasthenozoospermia is defined as the total number or concentration of spermatozoa and percentage of progressively motile spermatozoa, below the lower reference limits (total number < 39 × 10^6^ per ejaculate; concentration < 15 × 10^6^ per ml; progressively motile < 32%).”

The 5th centiles were used by mistake.
